# Recent advancements on innovative technologies and applications for non-destructive monitoring of tomato quality

**DOI:** 10.3389/fpls.2026.1931669

**Published:** 2026-09-28

**Authors:** Yirui Huang, Jiahao Gu, Mingyu Yang, Weifeng Zhang, Xuan Liu, Zhanmin Zhao, Xi Yang

**Affiliations:** 1 College of Information Engineering, Hebei Province Key Laboratory of Intelligent Sensing and Data Processing for Geoenvironment, Hebei Key Laboratory of Optoelectronic Information and Geo-detection Technology, Intelligent Sensor Network Engineering Research Center of Hebei Province, Shijiazhuang, China; 2 Medical Business Department, Frist Affiliated Hospital of Hebei Medical University, Shijiazhuang, China; 3 College of Future Information Technology, Shijiazhuang University, Shijiazhuang, China

**Keywords:** applications, data fusion (DF), multimodel, non-destructive techniques, tomato quality

## Abstract

Tomato quality assessment involves multiple external and internal attributes. Conventional reference methods remain important for accurate characterization but are often destructive, time-consuming, or unsuitable for repeated and online measurements. This review critically examines recent advances in nondestructive sensing technologies for tomato quality evaluation and organizes them according to sensing mechanism. Electromagnetic and optical techniques are assessed for their ability to characterize surface, compositional, spectral, and physiological information, while mechanical and tactile sensing approaches are discussed in relation to firmnes. Electrical and emerging sensors further extend quality assessment toward bioelectrical properties, volatile profiles, and other physicochemical changes. Particular attention is given to information fusion and multimodal sensing, with emphasis on the distinction between homogeneous spectral fusion and heterogeneous integration of physically complementary modalities. Future research should therefore prioritize complementary sensor selection, independent cross-condition validation, calibration transfer, interpretable modeling, system miniaturization, and standardized implementation in sorting, storage, and postharvest monitoring. In general, the transition from isolated laboratory models to transferable and operational quality-monitoring systems remains a central challenge for nondestructive tomato sensing.

## 1 Introduction

Tomato (*Solanum lycopersicum*) is an economically and nutritionally important horticultural crop that is widely consumed as a fresh and processed product. Tomato quality is closely associated with consumer acceptance, nutritional value, commercial grading, and postharvest management. It is characterized by multiple external and internal attributes, including color, size, shape, surface defects, firmness, soluble solids content (SSC), titratable acidity, pigments, and other biochemical components (Li et al., 2021; Mun et al., 2021). These quality attributes change continuously during tomato development, ripening, and postharvest storage. For example, pigment accumulation contributes to the characteristic color transition during ripening, while changes in cell-wall structure are accompanied by tissue softening and changes in firmness. Meanwhile, changes in soluble solids content (SSC), titrable acid content (TA), volatile compounds, and other biochemical constituents affect the flavor, nutritional characteristics, and commercial value of tomatoes (De Chiara et al., 2024; De Wit et al., 2024). The coexistence of these external and internal attributes, together with their dynamic changes during ripening and storage, makes reliable quality assessment an important requirement throughout tomato grading (Olorunfemi et al., 2024), distribution, and postharvest management (Parisi et al., 2021; Chen et al., 2024).

Conventional tomato quality assessment commonly relies on physicochemical analysis, mechanical measurements, sensory evaluation, and visual inspection. Physicochemical analyses can provide quantitative measurements of SSC, TA, pigments, and other chemical constituents, while mechanical measurements can directly characterize firmness and related properties (Rashvand et al., 2025). However, many of these methods require cutting, extraction, penetration, compression, or other forms of sample preparation and therefore may destroy or alter the tested object (Mohd Ali et al., 2023). Sensory and visual assessments are useful for evaluating appearance and market-related quality, but they can be affected by subjective judgment and provide limited information about internal properties (De Luna et al., 2019). These limitations are particularly relevant to commercial tomato production and postharvest handling, where large numbers of individual fruits need to be evaluated rapidly according to maturity, quality, and defects while maintaining their integrity (De Luna et al., 2020). Therefore, there is a growing need for rapid, objective, and reliable approaches that can assess multiple quality attributes of intact tomatoes without compromising their subsequent use. Nondestructive assessment has consequently become an important direction for improving the efficiency and scalability of tomato quality evaluation.

In response to these requirements, non-destructive sensing technologies have been increasingly developed for quality assessment of fruits and vegetables. Unlike conventional analytical methods that directly measure a specific quality attribute, non-destructive techniques generally detect physical, optical, electromagnetic, mechanical, acoustic, or chemical responses from intact biological tissues and subsequently establish relationships between the measured signals and quality-related characteristics (Akter et al., 2024; Huang et al., 2025a; Gajdoš Kljusurić et al., 2026). Machine vision and imaging techniques capture spatial variations in reflected, transmitted, or emitted signals and have been widely used to characterize appearance, color, shape, and surface defects (Bono et al., 2026). Spectroscopic techniques exploit wavelength-dependent interactions between electromagnetic radiation and biological tissues, including absorption, reflection, scattering, and emission, to obtain information associated with tissue composition and structure, while multispectral and hyperspectral imaging further combine spectral and spatial information (Du et al., 2020; Hernández-Hierro et al., 2022; Liu et al., 2024a). Mechanical, acoustic, and ultrasonic techniques measure responses such as deformation, force, vibration, or wave propagation associated with tissue structure and mechanical properties (De Ketelaere et al., 2006; Mizrach, 2007). Chemical and electrochemical sensing approaches, including electronic noses and related sensor systems, detect changes in volatile compounds, ionic species, or other chemical characteristics (Kim et al., 2023; Padilla et al., 2024). These technologies have expanded the range of information that can be obtained from intact fruits and vegetables and have provided important methodological foundations for non-destructive quality assessment.

The rapid diversification of non-destructive sensing technologies has gradually shifted tomato quality assessment from single-attribute or single-sensor measurement toward more comprehensive sensing of multiple quality characteristics (Li et al., 2026). Individual sensing modalities can provide valuable but inherently incomplete information. Imaging techniques can describe spatial variations in color, shape, and surface defects (Da Costa et al., 2020; De Araújo Gomes et al., 2025). Spectroscopy can provide information related to chemical composition and tissue characteristics (Hernández-Hierro et al., 2022; Hassan et al., 2025). Tactile and mechanical sensing can characterize firmness and tissue response (Mizrach, 2008), while chemical sensors can capture changes in chemical signatures (Kim et al., 2023). These differences reflect the distinct information domains accessible to different sensing mechanisms. For tomatoes, this complementarity is particularly relevant because ripening and postharvest deterioration involve simultaneous changes in appearance, texture, water status, chemical composition, and physiological condition. Consequently, recent research has increasingly explored combinations of imaging, spectroscopy, mechanical or tactile sensing, and other sensor modalities to obtain a more comprehensive representation of tomato quality. By integrating complementary features from multiple sensing modalities, multimodal approaches can provide more comprehensive information on tomato quality and have the potential to improve the accuracy of quality prediction compared with individual sensing modalities (Huang et al., 2024; Li et al., 2025, 2026).

The multidimensional nature of tomato quality provides a strong rationale for integrating heterogeneous sensing information. However, effective multimodal assessment requires appropriate strategies for integrating heterogeneous data acquired at different scales, dimensions, or sensing domains (Li et al., 2026). The increasing volume and complexity of nondestructive sensing data have also promoted the use of machine learning and deep learning for tomato quality assessment, particularly for high-dimensional spectral, imaging, and multimodal datasets (Pu et al., 2023; Li et al., 2024). These approaches can identify complex relationships between sensor responses and tomato quality attributes, but their predictive outputs may be difficult to interpret solely from model performance. Explainable artificial intelligence (XAI) applied to examine the contribution of spectral, spatial, mechanical, or chemical features to model predictions and to establish more interpretable relationships between sensing responses and tomato quality characteristics (Ahmed et al., 2025a; Contreras and Bocklitz, 2025; Ding et al., 2025). In multimodal models, XAI can further reveal the relative contributions and complementary roles of different sensing modalities, thereby helping to connect model predictions with physically meaningful sensing information (Shen et al., 2025). This capability is important for moving beyond prediction accuracy toward a more transparent and interpretable understanding of nondestructive tomato quality assessment.

Existing reviews have provided important foundations for understanding nondestructive sensing and intelligent analysis in tomato and other horticultural products (Huang et al., 2025a; Fu and Sun, 2026). However, the rapid diversification of sensing modalities and the increasing integration of heterogeneous sensing data make it necessary to examine tomato nondestructive quality assessment from a more integrated perspective (Du et al., 2020; Akter et al., 2024; Zhang et al., 2025b). In particular, different sensing technologies are often discussed separately, the transition from individual sensing to multimodal assessment raises important questions regarding information complementarity, data integration, and model interpretability that require critical discussion. This review aims to provide a critical overview of recent advances in non-destructive technologies for tomato quality assessment by connecting sensing mechanisms, measurable information, intelligent analysis, and practical applications. The major technologies are organized according to their underlying sensing mechanisms, including electromagnetic and optical techniques, mechanical and tactile sensing techniques and electrical and emerging sensing techniques. Their applications in tomato quality assessment are critically examined in terms of measurable quality attributes, sensing capabilities, advantages, limitations, and representative application scenarios. Multimodal sensing and data-fusion strategies are further analyzed in terms of information complementarity, fusion architectures and interpretability. Finally, current limitations and future requirements for transferable, standardized, and practically deployable tomato quality-monitoring systems are discussed.

## 2 Literature search strategy

A structured literature search was conducted using the Web of Science Core Collection, Scopus, and Google Scholar to identify studies published primarily between 2015 and 2026. Search terms combined’tomato’and ‘quality assessment’ or ‘nondestructive detection’ with terms describing major sensing approaches, including ‘spectroscopy’, ‘spectral imaging’, ‘RGB imaging’, ‘machine vision’, ‘mechanical sensing’, ‘tactile sensing’, ‘electrochemical’, and ‘electrical properties’. Additional searches using “multimodal sensing”, “sensor fusion”, and “data fusion” were performed to identify studies integrating complementary sensing information. Studies reporting sensing principles, analytical methods, experimental validation, or applications to tomato quality assessment were prioritized. Although the main search period began in 2015, representative classical studies published before 2015 were not excluded mechanically. Earlier studies were retained when they established important sensing principles, measurement methods, or analytical foundations. Reference lists of relevant reviews and primary studies were further screened to identify such publications. Highly relevant studies on other fruits and vegetables were also included selectively when they provided transferable evidence for understanding sensing principles, comparing methods, or discussing future research directions.

## 3 Tomato quality assessment

Tomato quality is a multidimensional and dynamic characteristic of the fruit.!!!The external qualities includes fruit size, surface color, and defects. The internal characteristics including firmness, SSC, TA, pigments, and other biochemical constituents (Nturambirwe and Opara, 2020). These quality attributes are closely associated with fruit growth and ripening and are influenced by coordinated changes in tissue development, pigment metabolism, cell-wall structure, carbohydrate and organic acid metabolism, and water status (Parisi et al., 2021). As a result, different quality attributes may change concurrently during ripening and may continue to evolve during postharvest storage, although their rates and patterns of change are not necessarily identical (De Wit et al., 2024). This section discusses tomato quality from external quality attribute and internal quality attributes, quality indicators and the corresponding traditional detection methods are summarized in Figure captions **Figure 1**.

**Figure 1:**
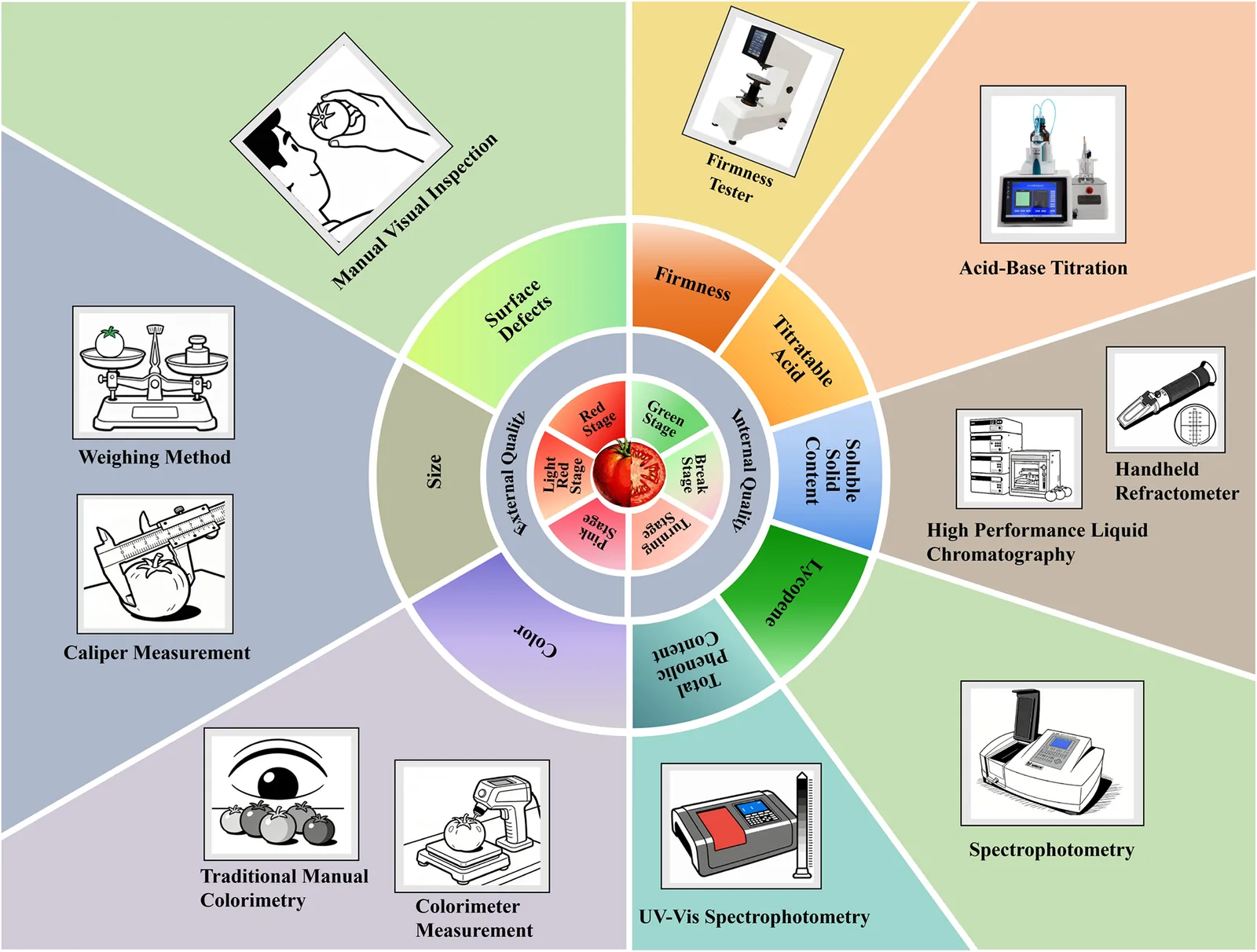
Tomato quality indicators and the corresponding traditional detection methods.

### 3.1 External quality attributes

External quality attributes are directly observable characteristics of tomato fruits and constitute important criteria for commercial grading, consumer acceptance, and postharvest handling. They mainly include color, size and visible defects. These attributes are influenced by fruit development, ripening, cultivation conditions, and postharvest handling, and therefore reflect different aspects of the physical state of the fruit.

Surface color changes substantially during tomato ripening and is closely associated with the transition from the mature-green stage to the red-ripe stage. As shown in **Figure 2**, during the fruit ripening process, chlorophyll is progressively degraded, whereas carotenoid accumulation, particularly lycopene accumulation, increases, resulting in a gradual transition of the peel from green to red (Alba et al., 2000; Lee et al., 2012). In tomato fruits, color development is also spatially heterogeneous, with coloration progressing across different regions of the fruit during ripening (Seymour et al., 2013). Thus, color provides not only a visual attribute for commercial grading but also an observable manifestation of physiological and biochemical changes occurring during fruit maturation.

**Figure 2:**
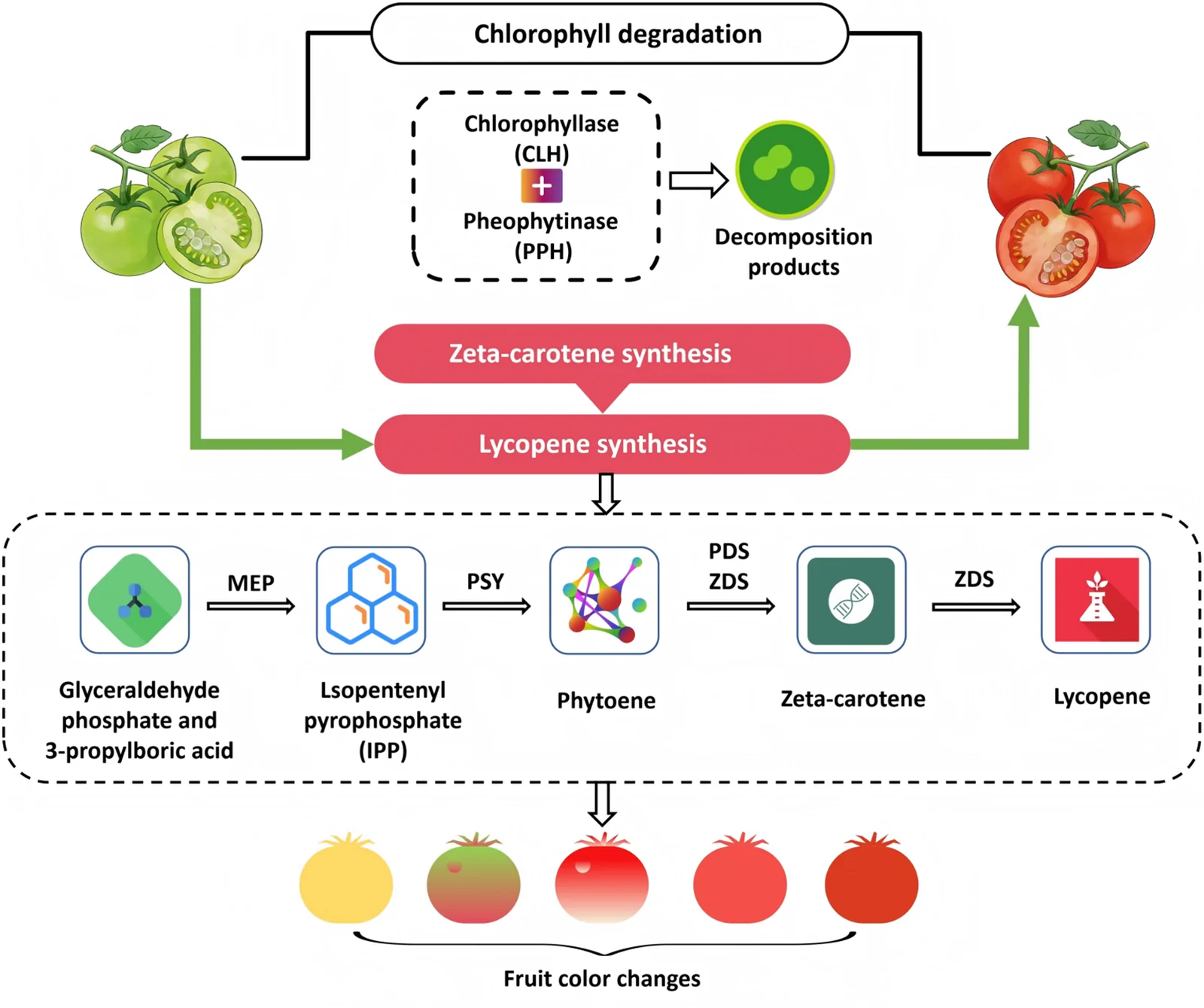
Schematic diagram of the synthesis and decomposition of lycopene and chlorophyll during the ripening of tomatoes. MEP, Methyl erythritol 4-phosphate; PSY, Phytoene synthase; PDS, Phytoene desaturase; ZDS, Zeta-carotenedesaturase.

Traditional color assessment is mainly based on visual comparison with standard color charts or instrumental color measurement. Visual assessment is simple and inexpensive but is affected by observer subjectivity, whereas colorimeters provide quantitative measurements using standardized color spaces such as CIELa^*^b^*^ (Piriya et al., 2017). However, point-based measurements has obvious limitation that cannot characterize the heterogeneity of the color distribution on the fruit surface.

Fruit size and shape are closely related to fruit growth and are important for harvesting, grading, and packaging. During fruit development, changes in fruit dimensions are associated with tissue growth and water accumulation, resulting in differences in fruit mass, diameter, and shape among developmental stages and cultivars. Conventional measurements generally rely on calipers and weighing, which are simple and inexpensive but require individual handling and are relatively inefficient for large numbers of fruits (Kondo, 2010).

Surface defects represent another major component of external quality. Tomato fruits may develop lesions, depressions, insect damage, cracks, scratches, or other defects during growth, harvesting, sorting, transportation, and storage (De Wit et al., 2024). Some defects remain localized at the surface, whereas mechanical injury may also affect tissues beneath the peel. Visual inspection is commonly used to assess these defects, but its accuracy can be influenced by observer experience and fatigue. Therefore, external quality encompasses both relatively stable geometric characteristics, such as size and shape, and dynamic surface characteristics associated with maturation and physical damage.

### 3.2 Internal quality attributes

Internal quality attributes describe the physical and biochemical characteristics of tomato tissues that cannot be fully determined from external appearance alone. Important indicators include firmness, soluble solids content (SSC), titratable acidity (TA), pigments such as lycopene, and total phenolic content (TPC) (Kaur et al., 2017). These attributes are closely associated with texture, flavor, nutritional value, maturity, and postharvest quality.

Firmness reflects the mechanical properties and structural integrity of tomato tissues and changes markedly during ripening. As ripening progresses, hydrolysis of pectic polysaccharides and degradation of intercellular adhesion contribute to the weakening of cell-wall structures, resulting in progressive tissue softening (Seymour et al., 2013). Therefore, firmness is closely related to maturity, handling tolerance, storage performance, and shelf life. Conventional firmness measurements commonly use puncture or compression-based methods (Nishinari and Fang, 2018). These approaches provide direct measurements of the mechanical response of tomato tissue but generally involve localized deformation or damage and may be affected by factors such as peel thickness (Li et al., 2016).

Changes in soluble solids and acidity are also important manifestations of tomato ripening. During maturation, carbohydrate metabolism contributes to changes in soluble sugars, while organic acid metabolism alters the acidity of the fruit (Carrari et al., 2006; Zhang et al., 2023). Water status may further influence the concentration of soluble constituents. Consequently, SSC and TA change with fruit development and contribute to the sugar–acid balance that strongly influences tomato flavor (Wang et al., 2023; Zhang et al., 2023). SSC is commonly determined using a refractometer, whereas TA is generally measured by acid–base titration. These methods are widely used as reference measurements because they provide quantitative physicochemical information, but they require juice or extracted samples and therefore cannot be repeatedly applied to the same intact fruit (Huang et al., 2021).

Tomato ripening is accompanied by changes in pigments and other bioactive compounds, including lycopene, carotenoids, phenolic compounds, and vitamin-related constituents (Seymour et al., 2013). These compositional changes contribute to the nutritional and functional characteristics of the fruit and occur concurrently with visible color development and other maturity-related changes (Fraser et al., 2009; Cheynier et al., 2013). Reference analysis of these constituents commonly involves spectrophotometric or chromatographic methods. Spectrophotometric measurements can quantify light-absorbing compounds (Elhamdaoui et al., 2024; Xu et al., 2025), whereas chromatographic methods provide separation and quantitative determination of individual compounds (Vidal-Casanella et al., 2022; Tsiasioti and Tzanavaras, 2024). Such approaches are valuable for obtaining reference values but generally involve extraction or other forms of sample preparation.

Tomato quality is a continuously evolving state characterized by the concurrent development of appearance, mechanical properties, and internal physicochemical composition. The progression of these attributes is influenced by the maturity at harvest and subsequent storage conditions, and their changes may show different temporal patterns. Therefore, a comprehensive description of postharvest tomato quality requires consideration of multiple quality attributes and their relationships rather than reliance on a single indicator.

## 4 Non-destructive techniques for tomato quality assessment

Non-destructive tomato quality assessment relies on sensing technologies that capture complementary information associated with external appearance, internal composition, physiological status, mechanical properties, and electrical responses. As shown in **Figure 3**, electromagnetic and optical techniques provide rapid access to surface and compositional information, while mechanical, acoustic, and tactile sensing characterize firmness and tissue response. Electrical and emerging sensors further extend detection toward bioelectrical properties and other physicochemical changes. These techniques differ in information obtained, spatial coverage and measurement conditions. The following section will explore their sensing characteristics and typical applications in the assessment of tomato quality.

**Figure 3:**
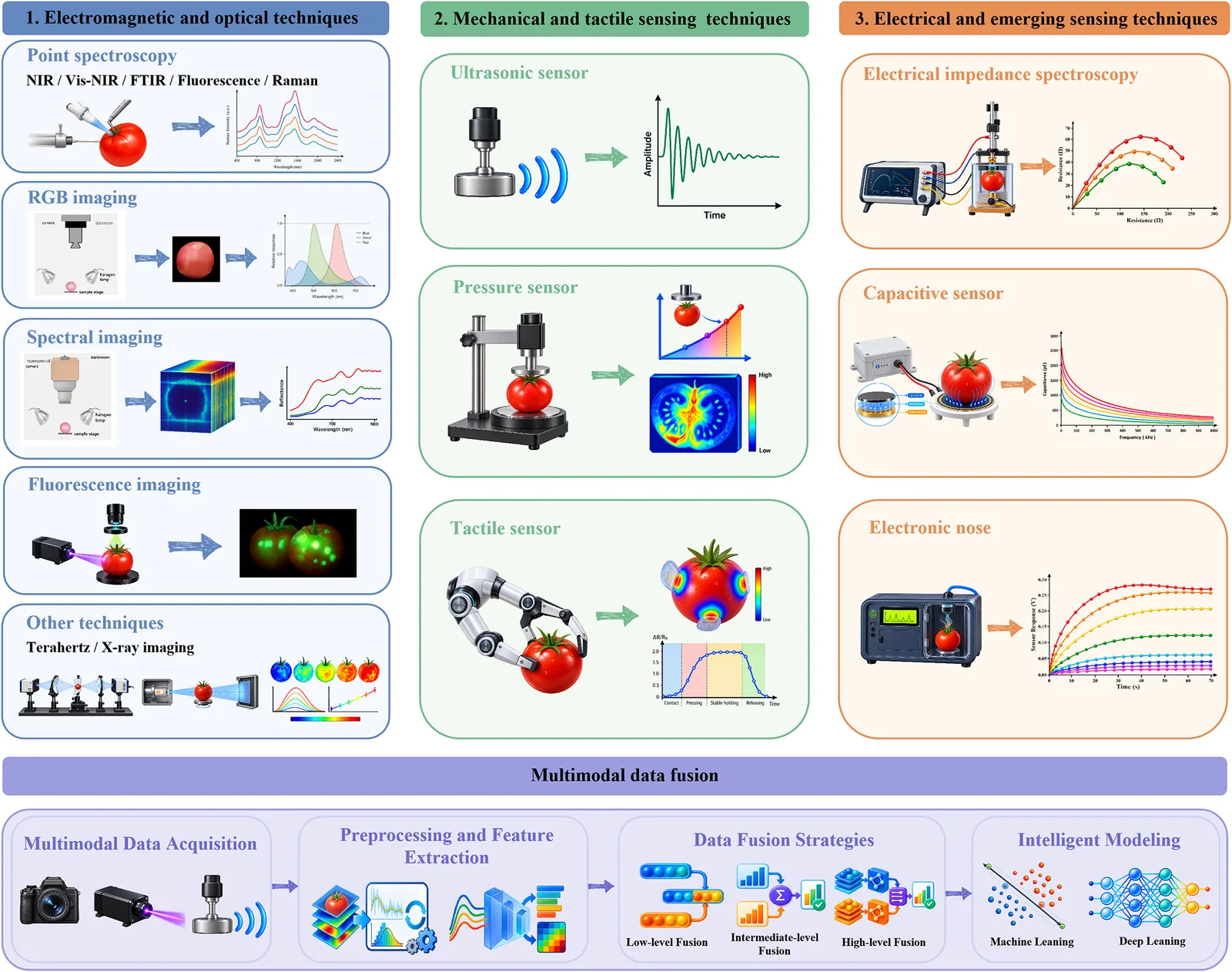
Classification of non-destructive technologies for tomato quality evaluation.

### 4.1 Electromagnetic and optical techniques

Electromagnetic and optical sensing constitutes the most extensively investigated group of nondestructive techniques for postharvest tomato quality assessment. These approaches rely on the interaction between electromagnetic radiation and tomato tissues, including absorption, reflection, scattering, transmission, fluorescence emission, and Raman scattering. Because pigments, water, organic constituents, and tissue structures exhibit wavelength-dependent optical responses, the resulting signals can provide indirect information about both external appearance and internal quality attributes (Xu et al., 2025; Gajdoš Kljusurić et al., 2026). Quality information is inferred from the statistical relationship between the measured signal and reference measurements (Xuesong et al., 2024).

Different electromagnetic and optical modalities provide different forms of information. Point spectroscopy primarily provides spectral information from a selected measurement region, whereas imaging-based techniques additionally preserve spatial information (Cozzolino, 2025). RGB imaging is mainly sensitive to visible surface characteristics, while multispectral and hyperspectral imaging extend the spectral dimension and can provide spatially resolved spectral information (Kondo, 2010; Huang et al., 2025a). Fluorescence and Raman spectroscopy provide additional information through emission and inelastic scattering processes, respectively (Slavova et al., 2023; Xu et al., 2023). Applications of electromagnetic and optical techniques in non-destructive quality monitoring of tomatoes are summarized in **Table 1**.

**Table 1:** Applications of electromagnetic and optical techniques in non-destructive quality monitoring of tomatoes.

Technology	Sample type	Parameters	Algorithm	Accuracy	References
NIR spectrometer	Processing tomato N Processing tomato E Cherry&cocktail	SSC	PLS	R^2^ = 0.72	(Ibáñez et al., 2019)
				R^2^ = 0.51	
				R^2^ = 0.87	
				R^2^ = 0.63	
				R^2^ = 0.92	
NIR spectrometer	Cherry tomato Currant tomato Non-cherry tomato	SSC	LS	R^2^ = 0.829	(Ino et al., 2023)
MicroNIR spectrometer	San Marzano tomato	Fresh weight	PLS	R^2^ = 0.61	(Castrignanò et al., 2019)
		Dry matter		R^2^ = 0.62	
		SSC		R^2^ = 0.57	
		TA		R^2^ = 0.53	
		PH		R^2^ = 0.51	
NIR spectrometer	Cherry tomato	SSC	BPNN	R^2^ = 0.8328	(Tan et al., 2022)
Vis-NIR spectrometer	——	SSC/TA	PLS	R^2^ = 0.801	(Tasioulas et al., 2025)
		PH		R^2^ = 0.780	
Vis-NIR spectrometer	Provence tomato	SSC	PLS	R^2^ = 0.912	(Cai et al., 2024)
Vis-NIR spectrometer	80 varieties of tomatoes	Lycopene	PLS	R^2^ = 0.85	(Duckena et al., 2023)
		Dry matter		R^2^ = 0.83	
Vis-NIR spectrometer	5 varieties of tomatoes	Dry matter	PLS	R^2^ = 0.59	(Arruda De Brito et al., 2022)
Hand-held Spectroradiometer	Harvested tomato	Firmness	Non-linear regression	R^2^ = 0.70	(Kumar et al., 2022)
	Stored tomato			R^2^ = 0.66	
	Harvested tomato	Total carotenoids	Non-linear regression	R^2^ = 0.86	(Kumar et al., 2023)
	Stored tomato			R^2^ = 0.69	
Handheld near infrared enhanced spectrometer	Red Rose tomato	Flesh firmness	PLS	R^2^ = 0.6790	(Alenazi et al., 2020)
		Lycopene		R^2^ = 0.8647	
		*ß*-carotene		R^2^ = 0.7082	
		TPC		R^2^ = 0.8343	
		Total flavonoid content		R^2^ = 0.7900	
FT-NIR spectrometer	Grape tomato Cherry tomato	SSC	PLS	R^2^ = 0.93	(Camps and Gilli, 2017)
		TA		R^2^ = 0.91	
		SSC/TA		R^2^ = 0.71	
		Texture parameters		R^2^ = 0.7-0.97	
ATR-FTIR spectroscopy	——	Fruit ripening stages	SVM	Acc = 99-100%	(Skolik et al., 2019)
Fluorescence spectroscopy	Green house tomato	Species identification	Multi-Class Classifier	Acc = 100%	(Slavova et al., 2023)
Raman spectroscopy	Cherry tomatoes	Freshness	DA	Acc = 85.6%	(Fu et al., 2016)
Handheld Raman Spectrometer	——	All-trans-lycopene	ANN	R = 0.9	(Akpolat et al., 2020)
			PLS	R = 0.87	
RGB imaging	Green tomatoes	Size classification	SVM	Acc = 95%	(De Luna et al., 2019)
			KNN	Acc = 92.5%	
			ANN	Acc = 92.5%	
RGB imaging	Cherry tomato	Mass	SVM	R^2^ = 0.9706	(Nyalala et al., 2019)
		Volume		R^2^ = 0.9694	
RGB imaging+Laser	Provence tomato	Diameter	YOLOv8s	MAPE =1.6%	(You et al., 2025)
		Volume		MAPE = 4.79%	
RGB imaging	Cherry tomato Heirloom tomato	Defect discrimination	RBF-SVM	Acc = 98.9%	(Ireri et al., 2019)
RGB imaging	Brazilian tomatoes	Grading	ResNet50	Acc = 94.6%	(Da Costa et al., 2020)
RGB imaging	——	Grading	SVM	Acc = 0.923-1	(Dhakshina Kumar et al., 2020)
RGB imaging	Large Tomato	Freshness recognition	ResNet34	Acc = 98.38%	(Huang et al., 2025b)
RGB imaging	——	Maturity grading	SVM	Acc = 99.81%	(De Luna et al., 2020)
RGB imaging	——	Maturity grading	GSBF-YOLO	Acc = 89.4%	(Hao et al., 2025)
RGB imaging	Cherry tomato	Grading	YOLOv4	Acc = 99.9%	(Cheng et al., 2022)
RGB imaging	Cherry tomato	Classifying of healthy and unhealthy	CNN-SVM	Acc = 93.54%	(Mputu et al., 2024)
RGB imaging	——	Maturity grading		Acc = 93.88%	(Pisharody et al., 2025)
RGB imaging	Cherry tomato	Classifying of healthy and unhealthy	PCA	Acc = 83.6%	(Fonseca et al., 2025)
Multispectral imaging	——	Lycopene content	BPNN	R^2^ = 0.938	(Liu et al., 2015)
		TPC		R^2^ = 0.965	
Hyperspectral imaging	Shifan 42	SSC	RNN	R^2^ = 0.88	(Zhao et al., 2023)
		Firmness		R^2^ = 0.92	
		TA		R^2^ = 0.87	
		Lycopene		R^2^ = 0.94	
Hyperspectral imaging	Cherry tomato	Firmness	ResNet	R^2^ = 0.532	(Xiang et al., 2022)
		SSC		R^2^ = 0.901	
Hyperspectral imaging	Cherry tomato	SSC	PLS	R^2^ = 0.95	(Ruizendaal et al., 2024)
Fluorescence imaging	——	Maturity classification	PCA	——	(Ma et al., 2024)
Fluorescence imaging	Green tomato	Maturity classification	——	Acc = 100%	(Fatchurrahman et al., 2020)
Fluorescence imaging	Momotaro	Quality detection	——	——	(Konagaya et al., 2020)

NIR, Near-infrared; Vis-NIR, Visible near-infrared; FT-NIR, Fourier transform near-infrared; ATR-FTIR, Attenuated total reflection Fourier-transform infrared; SSC, Soluble solid content; TA, Titratable acid; TPC, Total phenolic content; PLS, Partial least square; LS, Linear regression; BPNN, Back propagation neural networks; SVM, Support vector machine; DA, Discriminant analysis; ANN, Artificial neural networks; RBF-SVM, Radial basis function-support vector machine; CNN, Convolutional neural networks; SegLoRA, SegFormer architecture with the Low-Rank adaptation; PCA, Principal component analysis; RNN, Recurrent Neural Network; Acc, Accuracy; R, Correlation coefficient; R^2^, Coefficient of determination; MAPE, Mean absolute percentage error.

#### 4.1.1 Point spectroscopy

Point spectroscopy enables rapid and nondestructive assessment of tomato quality by relating wavelength-dependent optical responses to changes in fruit composition and tissue properties. Absorption, diffuse reflection, fluorescence, and inelastic scattering can provide information associated with water, soluble constituents, pigments, cell-wall components, and physiological status. Measured spectrum generally represents the combined response of several chemical and structural factors rather than the direct measurement of an individual quality attribute (Hernández-Hierro et al., 2022; Liu et al., 2024a). Near-infrared (NIR) and Visible Near-infrared (Vis-NIR) spectroscopy have received the greatest attention because they allow rapid measurements of intact fruit and can be implemented using portable instruments (Elhamdaoui et al., 2024; Cozzolino, 2025). Fourier-transform infrared spectroscopy, fluorescence spectroscopy, and Raman spectroscopy provide complementary information related to molecular composition or physiological state (Camps and Gilli, 2017; Bose et al., 2018; Xu et al., 2023). Point spectroscopy offers simple acquisition and data-processing requirements but provides limited information on spatial heterogeneity within individual fruit.

NIR and Vis-NIR spectroscopy have been investigated for SSC, TA, dry matter, firmness, pigments and maturity. However, the accumulated evidence indicates that prediction performance is strongly influenced by the population used for calibration rather than by spectral range alone. Ibáñez et al. (2019) investigated taste-related compounds in several tomato types, including processing, cherry and cocktail, midsized tomatoes, and tomato landraces. Models developed for individual varietal groups generally performed better than a general model combining different types of tomatoes. This finding indicates that variation in cultivar and fruit characteristics can become an important source of spectral variability and may affect the relationship between spectral response and quality attributes. Similar cultivar-dependent variation was observed by Ino et al. (2023). Studies covering postharvest ripening further showed that spectral responses change concurrently with firmness, pigments, and antioxidant-related properties, emphasizing that NIR measurements integrate multiple physicochemical changes occurring during ripening (Alenazi et al., 2020). Reflectance based approaches have also identified visible wavelengths associated with ripeness, firmness, and carotenoid-related traits, suggesting that relatively simple spectral indices may be useful for selected attributes under controlled measurement conditions (Kumar et al., 2022, 2023). Furthermore, the miniaturization of the instrument has enhanced the feasibility of portable measurements, which is highly valuable for on-site field testing of tomato quality (Castrignanò et al., 2019; Tan et al., 2022).

Extending measurements into the visible region allows Vis-NIR spectroscopy to combine pigment- and color-related information with NIR responses associated with internal composition (Tasioulas et al., 2025). Nevertheless, the spectral propagation geometric characteristics remain an important determinant of spectral quality. Cai et al. (2024) using full-transmission Vis-NIR spectroscopy for tomato SSC estimation, showed that changes in the optical propagation path affected the acquired spectra and subsequent model performance, demonstrating that instrument configuration and fruit orientation cannot be treated as secondary experimental details. Sample diversity is equally important when interpreting prediction statistics.

Arruda De Brito et al. (2022) evaluated intact tomatoes collected across different producing regions and planting periods. They used a portable Vis-NIR spectrometer covering 280–1200 nm and investigated five tomato varieties. All samples were measured for dry matter and the training set data with a sample standard deviation of 0.955% was obtained. The prediction model for dry matter using spectral reflection values ranging from 729 to 975 nm, achieving R^2^ value of 0.59. In contrast, Duckena et al. (2023) investigated a much larger and more diverse collection comprising 80 tomato varieties using a Vis-NIR spectrometer covering 350–2500 nm, and the training set data for modeling was have a sample standard deviation of 1.356%. Prediction model performed R^2^ = 0.83 for dry matter. The two studies differed simultaneously in spectral coverage, number and diversity of tomato varieties and target sample population. The difference in R^2^ values represents the combined effect of spectral information and experimental sample variability. In particular, a higher prediction accuracy is not only related to a wider spectral range, but also depends on the distribution of the reference values.

Other point-spectroscopic approaches provide more specific biochemical or physiological information, although their practical scope differs from that of NIR and Vis-NIR spectroscopy. The Fourier transform Infrared (FTIR) spectrometer have been applied to texture, quality, and developmental characterization. Camps and Gilli (2017) used FTIR spectroscopy to monitor local and global texture-related properties during tomato shelf life, whereas Skolik et al. (2019) applied portable Attenuated total reflection Fourier-transform infrared (ATR-FTIR) spectroscopy and chemometrics to distinguish developmental and ripening stages of intact fruit. Notably, the latter study acquired spectra from multiple positions around each fruit, illustrating the need to account for spatial variability in fruit even when a point-based instrument is used. Fluorescence spectroscopy provides a different source of information because chlorophyll fluorescence and related parameters reflect physiological changes during ripening. Kasampalis et al. (2020) showed that selected fluorescence parameters could discriminate maturity stages and retain information on initial harvest maturity during storage even when surface color became less informative. Machine learning analysis of fluorescence spectra has also produced high classification accuracy for tomato variety discrimination (Slavova et al., 2023), but results obtained from a restricted number of cultivars should not be interpreted as evidence of equivalent performance under broader biological and environmental variation. Fluorescence therefore appears useful as a complementary indicator of physiological status, while the mechanistic links between fluorescence responses and broader postharvest quality deterioration remain an area requiring further investigation (Ma et al., 2024).

Raman spectroscopy offers greater molecular specificity through vibrational fingerprints and has mainly been investigated for carotenoid-related traits and freshness in tomatoes (Xu et al., 2023). Fu et al. (2016) developed a miniaturized 785 nm Raman system for intact tomatoes and obtained useful freshness discrimination, whereas quantitative prediction of lycopene was less satisfactory. In contrast, Akpolat et al. (2020) examined 106 samples covering different carotenoid profiles using handheld 1064 nm Raman spectroscopy and obtained effective discrimination of carotenoid groups and useful prediction of lycopene. The difference between these studies should not be attributed solely to the modeling algorithm because they also differed in sample composition, carotenoid range, excitation wavelength, and reference design. Rather, they demonstrate a broader requirement of quantitative spectroscopy: calibration samples must adequately represent the biological range of the target constituent before model performance can be meaningfully evaluated.

Point spectroscopy offers rapid acquisition, relatively simple instrumentation, and strong potential for portable tomato quality assessment, but the literature also exposes several limitations that are sometimes obscured by high dataset prediction statistics. Tomatoes are spatially and biologically heterogeneous. Consequently, random division of samples collected within a single experiment may evaluate interpolation within a specific population rather than true model transferability. Future studies should therefore place greater emphasis on independent external validation, multi-cultivar and multi-season sampling, standardized measurement geometry, calibration transfer between instruments, and explicit reporting of the reference-value distribution. Moreover, the principal advantage of point spectroscopy is operational simplicity and rapid measurement, whereas its main limitation is restricted spatial representation, providing a clear rationale for the development of spectral imaging approaches that combine spectral and spatial information.

#### 4.1.2 RGB imaging

Spectroscopy provides spectral information from a selected measurement region but does not preserve the spatial distribution of quality related signals across the fruit. Imaging technology combines spectral analysis with image visualization, achieving an improvement from “point” measurement to “surface” observation, addressing the limitation by combining electromagnetic sensing with spatial information. RGB imaging is the most accessible modality because of its relatively simple hardware, low cost, and compatibility with automated sorting systems. It primarily captures visible surface characteristics, including color, size, shape, texture, and external defects. In tomato postharvest assessment, these characteristics are directly relevant to grading, maturity classification, freshness evaluation, and defect screening.

Early RGB studies generally relied on explicitly defined color, geometric, and texture descriptors followed by conventional statistical or machine learning models. This strategy was effective when the target attribute had a clear visual or geometric basis. Nyalala et al. (2019) extracted two and three dimensional features from cherry tomato images and demonstrated that image derived geometry could support volume and mass estimation, although the relationship between these properties may vary with fruit density and internal structure. Similarly, De Luna et al. (2019) compared thresholding, conventional machine learning, and deep learning for tomato size classification and showed that increased model complexity did not necessarily improve prediction when the available dataset was limited. While color, texture, and shape descriptors were combined for automated grading, maturity assessment, and defect recognition (Ireri et al., 2019; Dhakshina Kumar et al., 2020). More recent feature extraction approaches have combined multiple color spaces to enhance visual differences associated with surface smoothness and wrinkling (Huang et al., 2025b). Collectively, these studies established the feasibility of RGB imaging for quality attributes that are directly expressed at the fruit surface, while also demonstrating its dependence on appropriate feature representation and controlled image acquisition.

Deep learning has reduced the dependence on manually designed descriptors by learning hierarchical visual representations directly from images. De Luna et al. (2020) incorporated convolutional object detection into tomato growth monitoring for fruit and flower recognition and combined it with maturity classification, illustrating the transition from isolated fruit inspection toward *in situ* monitoring. Subsequent studies have increasingly addressed environmental variability rather than only optimizing classification under controlled backgrounds. Large image collections have been used to distinguish healthy and defective tomatoes and to reduce confusion between true defects and visually similar structures such as calyxes and stem scars (Da Costa et al., 2020). Increasing dataset diversity and background complexity has also been investigated as a strategy to reduce overfitting and improve robustness (Cheng et al., 2022). In addition, lightweight and attention-enhanced architectures further target practical constraints such as leaf occlusion, overlapping fruits, variable scale, and computational cost (Mputu et al., 2024; Hao et al., 2025; Pisharody et al., 2025; You et al., 2025). These developments suggest that the principal contribution of deep learning is not simply higher classification accuracy, but an increased capacity to represent complex visual variation. Nevertheless, model performance remains closely dependent on the diversity of training data and the correspondence between training and deployment environments.

As RGB-based models become more complex, understanding the visual evidence underlying their predictions has emerged as an additional requirement. This is particularly relevant for maturity detection because color transitions, foliage, illumination, and background objects may produce correlated image patterns that allow high predictive performance without necessarily ensuring that the model relies on fruit specific features. Some studies have incorporated explainable artificial intelligence (XAI) to examine spatial feature responses. As illustrated in **Figure 4A**, Rahim et al. (2026) applied Gradient-Weighted Class Activation Mapping (Grad-CAM) to compare baseline YOLOv8m with the Tomato-Adaptive Attention model TAA-YOLOv8m across the Cross-Regional Tomato, Laboro Tomato, and Tomato Plantfactory datasets. The improved model produced more localized activation over tomato regions and reduced responses to surrounding backgrounds, indicating that its quantitative improvement was accompanied by more spatially focused feature representations. A complementary example is shown in **Figure 4B**, Cheng et al. (2026) visualized Grad-CAM responses of an improved YOLOv11n model under normal illumination, blur, backlighting, low light, and branch or leaf occlusion. The visualizations showed changes in feature localization after the introduction of illumination enhancement, noise suppression, and attention modules, providing qualitative evidence for how individual architectural modifications influenced model responses under challenging greenhouse conditions. In addition, Local Interpretable Model-agnostic Explanations (LIME) is also a method used for model interpretability analysis. Although it is applied to the representation of classification decision features in the assessment of avocado ripeness and quality (Lee et al., 2025), it demonstrates the extensive potential of XAI in evaluating quality models based on images.

**Figure 4:**
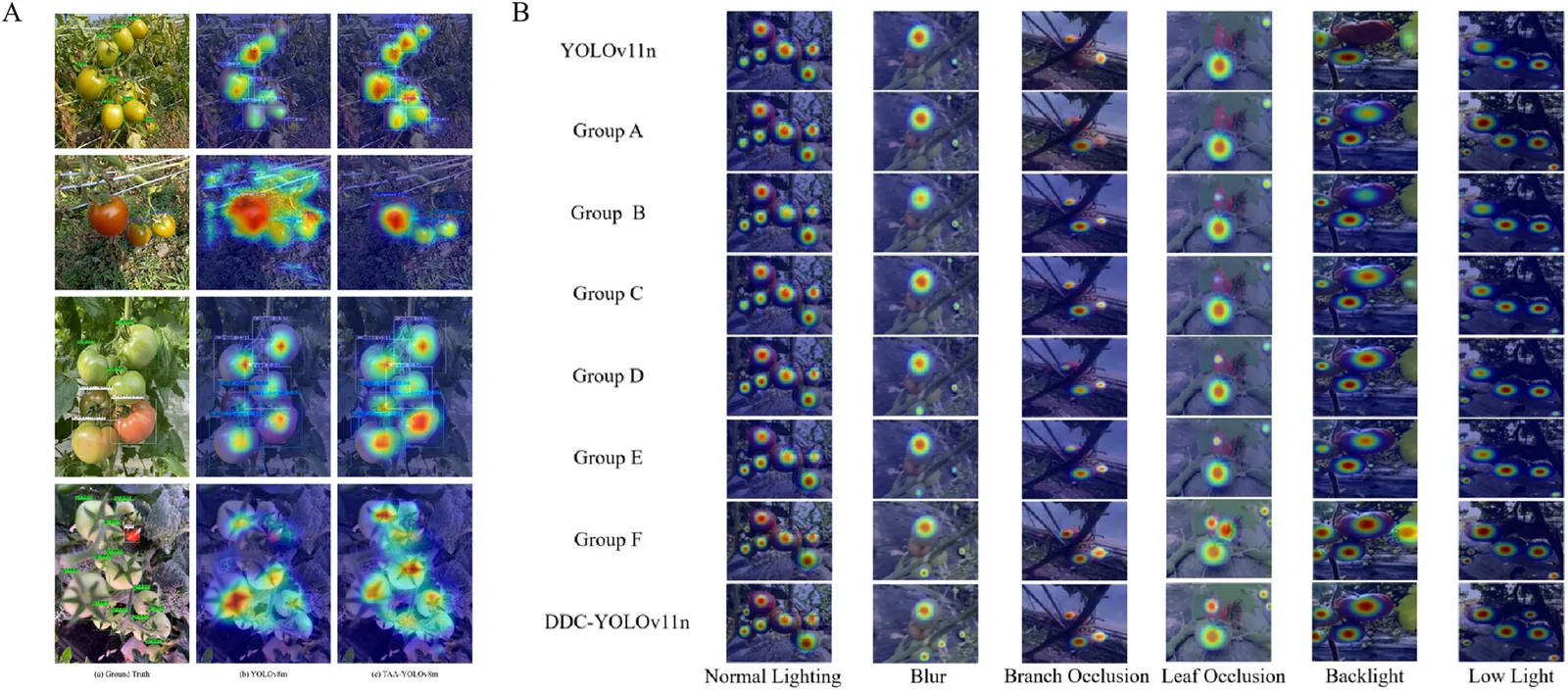
Comparison of the visualization results of different models used for detecting tomato ripeness. **(A)** Grad CAM visualizations of YOLOv8m (middle) and TAA- YOLOv8m (right) on representative examples. The (left) column shows original images with ground truth annotations. Rows 1–2 represent the Cross Regional Tomato dataset, row 3 the Laboro Tomato dataset, and row 4 the Tomato Plantfactory dataset. TAA exhibits more localized activation over fruit regions with reduced background responses compared to the broader activations observed in the baseline model. Reproduced from (Rahim et al., 2026), copyright 2026 with permission from Agriculture. **(B)** Grad CAM visualizations of YOLOv11n, DDC YOLOv11n and ablation experiment models across various scenarios. Reproduced from (Cheng et al., 2026), copyright 2026 with permission from Frontiers in Plant Science.

The increasing use of XAI also requires cautious interpretation. Grad-CAM and related saliency methods indicate image regions associated with model outputs, but localized activation over a tomato does not by itself establish a causal relationship between the highlighted feature and physiological maturity or quality. More broadly, RGB imaging remains fundamentally constrained by the information available in the visible surface phenotype. Illumination, background composition, occlusion, camera characteristics, cultivar-specific morphology, and maturity-related color differences can all alter image distributions, while internal biochemical attributes can only be inferred indirectly when they covary reliably with visible features. Therefore, RGB imaging is particularly effective for rapid and spatially resolved assessment of external tomato quality, whereas spectroscopy and multimodal sensing remain important when direct information on internal physicochemical properties is required.

#### 4.1.3 Multispectral imaging

Spectral imaging combines spectral measurement with spatial imaging, enabling spectral responses to be spatially resolved across the fruit. According to the number and continuity of acquired wavelengths, spectral imaging can generally be divided into multispectral imaging (MSI) and hyperspectral imaging (HSI). MSI acquires spatially resolved information at a limited number of selected wavelengths (Huang et al., 2025a). Because fewer spectral bands are acquired, MSI can reduce data volume and simplify acquisition and processing compared with HSI, although its performance depends strongly on whether the selected wavelengths contain sufficient information related to the target attribute (Castillo-Girones et al., 2024). In tomato quality assessment, Liu et al. (2015) demonstrated the feasibility of MSI for simultaneous nondestructive prediction of lycopene and total phenolic contents in intact fruits. Partial least squares (PLS), least squares-support vector machines (LS-SVM) and back propagation neural network (BPNN) models were compared, and BPNN achieved the best prediction performance, with R^2^ values of 0.938 and 0.965 for lycopene and total phenolics, respectively. These results demonstrated that selected multispectral information can provide sufficient information for quantitative assessment of specific biochemical attributes when appropriate spectral bands and modeling methods are used.

More studies have increasingly shifted the focus of MSI development from demonstrating detection feasibility to reducing model complexity. For example, Mlynárik et al. (2026) investigated waveband optimization for multispectral estimation of tomato lycopene concentration using a system covering 15 wavelengths from 380 to 940 nm. A six-band configuration combined with a shallow neural network achieved an R^2^ of 0.959, while a two band configuration retained an R^2^ of 0.951. These results showed that substantial spectral reduction may be feasible when the target attribute is clearly defined and informative wavelengths can be identified. Therefore, there is an important potential advantage of MSI for developing task-specific sensing systems with reduced hardware and computational requirement.

The trend of simplified and application-oriented optical sensing is also gradually emerging to reduce system costs. Sherafati et al. (2022) combined smartphone imaging with concentrated light-beam injection at selected wavelengths to predict several tomato quality indices. Different wavelengths or wavelength combinations were selected for different attributes, as 650 nm was used for carotenoid estimation, 532 and 650 nm were combined for lycopene and elasticity-related prediction, and 650 and 780 nm were used for total chlorophyll. This research provides a new direction for practical applications that the combination of improved RGB imaging technology and mobile computing aims to reduce system complexity and enhance portability.

From the analysis of the literature, it can be observed that recent studies have explored whether selected wavelengths can retain sufficient information for specific quality attributes while reducing acquisition, processing, and hardware requirements. However, this advantage is dependent on fixed tasks, because spectral information that is sufficient for one attribute may not adequately characterize another.

#### 4.1.4 Hyperspectral imaging

HSI integrates conventional imaging with spectroscopy by acquiring a spatially resolved spectrum for each pixel within a scene. Compared with RGB imaging, which primarily characterizes visible appearance, and point spectroscopy, which provides spectral information without preserving spatial heterogeneity. HSI enables the simultaneous assessment of spatial and spectral variations associated with fruit development and quality. During ripening process, tomato quality changes involve coordinated alterations in pigmentation, water status, soluble components, acidity, and tissue structure (Giovannoni, 2007; Seymour et al., 2013). These processes alter light absorption and scattering in different spectral regions and therefore provide the physicochemical basis for nondestructive assessment of maturity and internal quality attributes (Feng and Sun, 2012; Lorente et al., 2012).

The spectral response of tomato fruit should be interpreted in relation to the underlying biochemical and structural transformations rather than treated solely as a high dimensional spectral information. In the visible region, tomato ripening is accompanied by chlorophyll degradation and carotenoid accumulation, particularly the progressive accumulation of lycopene, producing pronounced changes in reflectance associated with the transition from green to red fruit. Chlorophyll absorption in the blue and red regions and the transition toward the red edge therefore provide information relevant to maturation (Alba et al., 2000). Fass et al. (2025) using spectra acquired from five tomato cultivars between 400 and 1000 nm, demonstrated substantial cultivar-dependent spectral variation while retaining recognizable absorption features associated with pigments and water. Their spectral profiles, summarized conceptually in **Figure 5A**. Beyond the visible region, near-infrared responses provide complementary information on internal composition. Absorption near approximately 970 nm is strongly influenced by O–H overtones and is therefore sensitive to water-related variation, whereas longer near-infrared regions contain overlapping O–H and C–H overtone and combination bands associated with water and organic constituents. These features are relevant to SSC, sugar and acid estimation but should not be interpreted as unique absorption markers for individual quality traits (Lorente et al., 2012; Ding et al., 2026).

**Figure 5:**
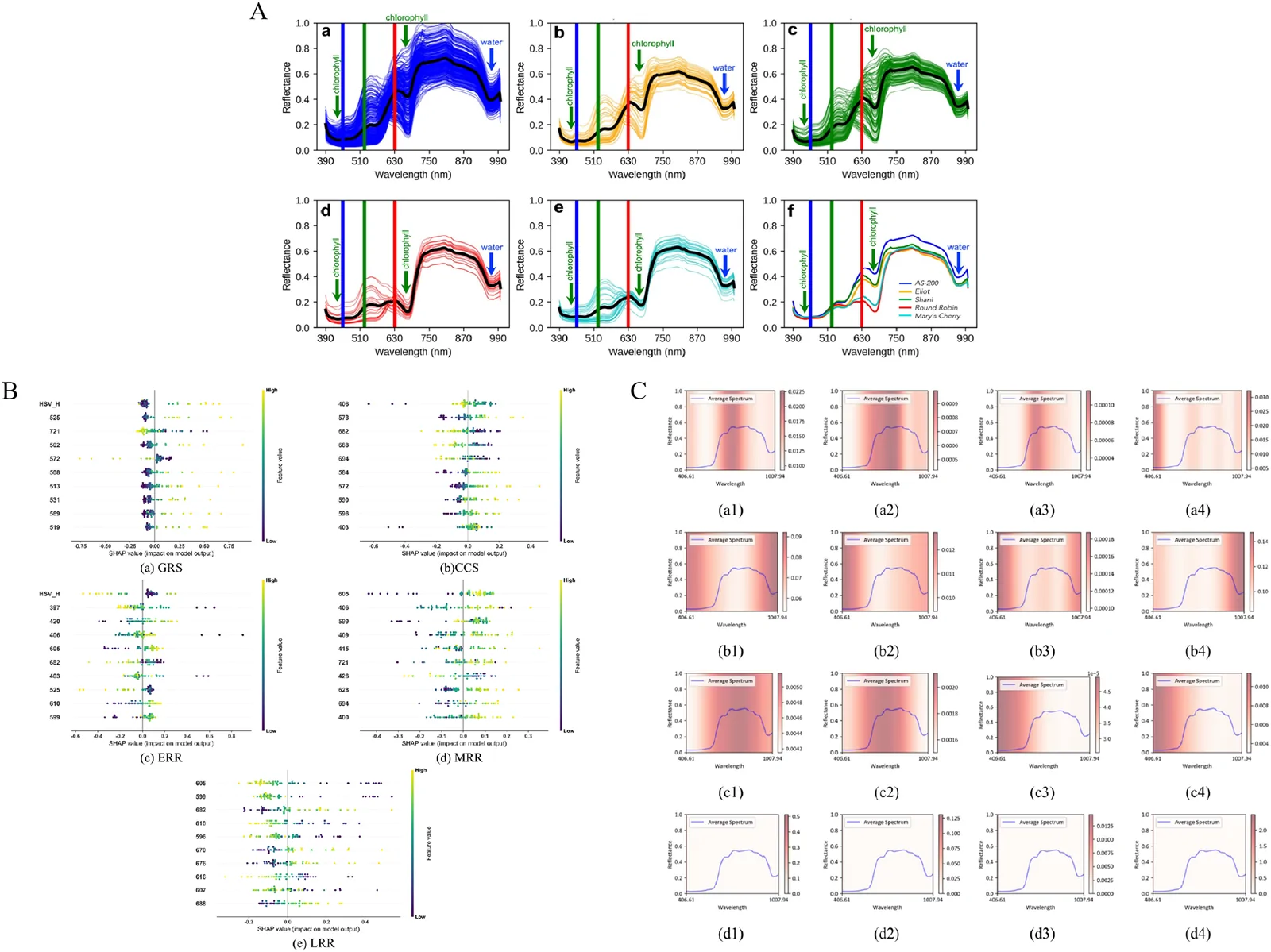
Tomato spectral data analysis based on interpretable artificial intelligence. **(A)** Fruit reflectance spectral of five tomato cultivars. **(a–e)** AS-200, Eliot, Shani, Round Robin, and Mary’s Cherry, respectively; **(f)** comparison of the mean spectra of the five cultivars. Reproduced from (Fass et al., 2025), copyright 2025 with permission from Computers and Electronics in Agriculture. **(B)** SHAP feature importance across different tomato ripening stages. **(a–e)** Green-ripe (GRS), colour-changing (CCS), early-red-ripe (ERR), medium-red-ripe (MRR), and late-red-ripe (LRR) stages, respectively. Reproduced from (Zhou et al., 2026), copyright 2026 with permission from Journal of Food Measurement and Characterization. **(C)** Grad CAM visualizations for tomato quality prediction using visible and near infrared spectral. **(a1–d4)** represent combinations of four models and four quality attributes. **(a–d)** VGG, ResNet, DenseNet, and Transformer, respectively; (1–4) soluble solids content (SSC), acidity, sugar content, and firmness. Reproduced from (Wang et al., 2025b), copyright 2025 with permission from Applied Food Research.

This physicochemical basis has enabled HSI to move beyond external maturity classification toward simultaneous estimation of multiple tomato quality attributes. Early and conventional chemometric approaches generally relied on spectral preprocessing, wavelength selection and regression or classification methods. Dai et al. (2023) combined competitive adaptive reweighted sampling with HSI and showed that reduced characteristic wavelengths could classify six tomato maturity stages with an accuracy of 95.83%, while an SVR model predicted lycopene content with R^2^ of 0.9652. Importantly, HSI also enabled spatial visualization of estimated lycopene distribution, illustrating an advantage that cannot be obtained from point spectroscopy alone.

More studies have extended this framework toward multi-trait and deep-learning analysis. Zhao et al. (2023) used HSI over 980–1660 nm to simultaneously assess firmness, SSC, lycopene, TA and to distinguish three maturity stages of processing tomatoes. Recurrent neural networks outperformed RF and PLS models for the investigated dataset, with R^2^ values exceeding 0.87 for quality prediction, while contribution analysis was used to identify wavelengths important to individual predictions. Xiang et al. (2022) similarly demonstrated the capacity of one-dimensional residual convolutional networks to model spectral sequences, reporting R^2^ = 0.901 for SSC prediction. However, their substantially lower performance for firmness (R^2^ = 0.532) highlights an important limitation that the model which yielded high performance for chemically related attributes cannot automatically be generalized to mechanically defined properties. Differences in penetration depth, spectral range, tissue heterogeneity and sample composition can all contribute to such variation among target traits and studies (Akter et al., 2024). The applications of deep learning changes the central question in HSI analysis from whether a model can accurately predict tomato quality to whether its predictions are based on spectrally and biologically plausible information (Contreras and Bocklitz, 2025).

XAI provides an additional layer of model evaluation by quantifying how individual wavelengths or spectral regions contribute to predictions (Arrighi et al., 2026). In this context, XAI is not merely a visualization tool, but can reveal whether different quality traits and maturity categories rely on distinct spectral information and identification of a limited number of informative wavelengths can guide dimensionality reduction and the subsequent design of lower-cost multispectral devices (Ahmed et al., 2025a).

Recent studies provide representative examples of these functions. Zhou et al. (2026) combined hyperspectral characteristic wavelengths with hue information and an attention-enhanced 1D-CNN for tomato ripeness detection. The 1D-CBAMCNN achieved 95.18% accuracy, approximately two percentage points higher than the baseline 1D-CNN, and SHAP analysis was subsequently used to quantify the contribution of spectral features. As illustrated conceptually in **Figure 5B**, the distribution of wavelength importance varies among maturity-related sample categories rather than remaining uniform across the ripening process. This observation is physiologically reasonable because the relative contributions of chlorophyll loss, carotenoid accumulation and other compositional changes evolve during ripening. More importantly, SHapley Additive exPlanations (SHAP) provides a means of testing whether the classification model follows these systematic spectral changes rather than relying exclusively on an opaque combination of correlated variables.

Grad-CAM provides a complementary explanation for deep spectral networks by identifying regions to which a trained architecture assigns high predictive relevance. Wang et al. (2025b) compared Grad-CAM responses from VGG, ResNet, DenseNet and Transformer models for SSC, acidity, sugar content and firmness. The resulting attention maps, summarized in **Figure 5C**, showed that model attention was not distributed uniformly over the spectrum and that different prediction targets could emphasize different spectral intervals. Such results are significant because agreement between model attribution and known chemical information increases confidence that the network has learned meaningful spectral relationships. At the same time, disagreement among architectures or instability of important wavelengths across datasets should not automatically be interpreted as model failure, because broad and overlapping absorption bands, collinearity and indirect correlations among tomato quality attributes can lead to multiple spectrally plausible solutions (Bai et al., 2025). XAI outputs should be interpreted together with fruit physiology and spectroscopy rather than used as independent proof of causality.

HSI has progressed from demonstrating spectral differences among maturity stages toward quantitative multi-attribute prediction, deep spectral learning and explainable modeling. However, greater practical value will depend on linking model-important wavelengths to tomato physiology, establishing robustness under independent environmental and biological variation, and translating reproducible spectral information into compact sensing systems (Arrighi et al., 2026). The role of XAI is particularly relevant in this transition. It can identify whether highly performing models exploit chemically meaningful information, reveal trait-specific spectral mechanisms and support rational wavelength reduction. These results can subsequently support development of snapshot multispectral cameras, filter-based imaging systems or compact spectrometers containing only wavelengths required for a specified quality task. These developments are essential if spectral imaging assessment of tomato quality is to progress from experimental validation toward reliable preharvest monitoring and automated postharvest quality control (Vignati et al., 2023).

#### 4.1.5 Fluorescence imaging technology

Fluorescence imaging records light emitted from endogenous fluorophores following optical excitation and can therefore provide physiological information that is not fully represented by conventional reflectance or RGB images (Rahman et al., 2017; Fang et al., 2023). In tomatoes, ripening alters chlorophyll, carotenoids, and fluorophores associated with surface tissues, resulting in measurable changes in fluorescence characteristics (Hoffmann et al., 2015). Fluorescence measurements have consequently been explored for monitoring tomato development and postharvest ripening. Its main relevance to nondestructive quality assessment lies in detecting maturity or storage related changes when differences in visible appearance are limited.

A representative application is the discrimination of visually similar green-stage tomatoes. Fatchurrahman et al. (2020) used hyperspectral fluorescence imaging with 365 nm excitation to distinguish mature green from immature green tomatoes. Although both groups exhibited similar external color, fluorescence around 690 nm differed between maturity stages and supported effective classification. The importance of this result lies in demonstrating that fluorescence can reveal maturity related physiological differences before they are clearly expressed in RGB appearance. This observation is consistent with earlier studies showing systematic changes in tomato fluorescence during ripening and the potential of fluorescence indices for monitoring maturity under preharvest and postharvest conditions (Hoffmann et al., 2015). Fluorescence imaging therefore provides a spatial extension of such measurements and can complement color-based maturity assessment.

Fluorescence responses may remain informative after tomatoes reach the red stage. Konagaya et al. (2020) monitored red tomatoes during nine days of storage using ultraviolet-induced visible fluorescence imaging and found that color derived responses changed mainly during the early storage period, whereas fluorescence-derived signals continued to vary thereafter. Similarly, chlorophyll fluorescence parameters have been reported to retain information related to harvest maturity after fruits become uniformly red, when conventional color measurements become less discriminative (Kasampalis et al., 2020). Recent work has further extended fluorescence assessment from signal intensity to temporal decay characteristics. Ma et al. (2024) developed a time-resolved laser-induced fluorescence method and observed a progressive decrease in fluorescence lifetime during storage of mature tomatoes. Fluorescence lifetime showed greater stability than fluorescence intensity for detecting early quality deterioration, suggesting that temporal information may complement conventional intensity-based measurements. However, current evidence for lifetime assessment remains limited and requires validation across broader cultivar and storage conditions.

Fluorescence imaging provides a useful complement to RGB and spectral imaging by capturing physiological changes associated with tomato maturity and postharvest deterioration. Future research should focus on identifying the biological origin of informative fluorescence signals Fu et al. (2025), standardizing acquisition conditions, and validating models across cultivars, seasons, and postharvest environments.

#### 4.1.6 Other technologies

Beyond the methods discussed above, emerging electromagnetic techniques provide additional information domains that may complement conventional tomato quality assessment. Terahertz (THz) spectroscopy and spectral imaging are sensitive to dielectric responses associated particularly with water and other polar constituents and have been investigated for nondestructive analysis of food dehydration, storage, freezing, fermentation, and related processes (Fu et al., 2024). Their potential relevance to fresh produce lies primarily in monitoring moisture-related and physicochemical changes that may not be fully characterized by visible or NIR measurements. However, evidence for intact tomato assessment remains limited. In particular, the high water content of fresh fruit can produce strong THz absorption, while system cost and interference from other matrix constituents remain practical constraints.

X-ray imaging provides a direct route to internal structural characterization because of its greater penetration depth. Wang et al. (2017) applied X-ray imaging to fresh cherry tomato and simultaneously obtained absorption, refraction, and scattering contrast. The scattering image revealed seed distribution more clearly than conventional absorption contrast, demonstrating that complementary X-ray contrasts can provide information on internal structures that is difficult to obtain using surface-sensitive optical imaging. Nevertheless, the complexity and cost of grating-based systems, together with the use of ionizing radiation and the limited number of tomato-specific studies, currently restrict their suitability for routine high-throughput quality monitoring.

Electromagnetic and optical methods reviewed in this section. Point spectroscopy provides localized compositional signatures, RGB imaging characterizes spatially resolved external phenotypes, multispectral and hyperspectral imaging integrate spatial and spectral information, fluorescence imaging adds excitation-dependent physiological responses and emerging THz and X-ray methods extend sensing toward additional physicochemical and internal structural information. Across these modalities, the major challenge is shifting from demonstrating feasibility under controlled conditions to establishing robust performance across cultivars, maturity stages, production environments, instruments, and postharvest conditions. Future technology selection should be guided by the measurement practicality, and validation under realistic conditions.

### 4.2 Mechanical and tactile sensing

Mechanical and tactile sensing provides a complementary route to optical methods by directly or indirectly characterizing the mechanical response of tomato tissues. Firmness decreases substantially during ripening and postharvest softening and is therefore closely related to handling tolerance, maturity, and consumer perception (Sirisomboon et al., 2012). Mechanical sensing approaches infer this property from fruit vibration, acoustic-wave propagation, contact force, or local deformation rather than from reflected or emitted electromagnetic signals (García-Ramos et al., 2005). Earlier studies focused primarily on whole-fruit acoustic and ultrasonic responses, whereas recent developments increasingly integrate compliant tactile sensors with soft robotic grippers, allowing mechanical quality information to be acquired during grasping and manipulation. Applications of electromagnetic and optical techniques in non-destructive quality monitoring of tomatoes are summarized in **Table 2**.

**Table 2:** Applications of Mechanical and tactile sensing technologies in non-destructive quality monitoring of tomatoes.

Technology	Sample type	Parameters	Algorithm	Accuracy	References
Ultrasonic tests	——	Firmness	Linear regression	R^2^ = 0.9157	(Mizrach, 2007)
GelSight sensor	Cherry tomato	Firmness	CNN	R^2^ = 0.6818	(He et al., 2024)
Fin ray gripper	——	Firmness	CNN-LSTM	R^2^ = 0.795	(Lin et al., 2023)
		Maturity grading	LSTM	Acc = 84.6%	
Hand-shaped gripper	——	Maturity grading	Algorithm	Acc = 99%	(Liu et al., 2025b)

R^2^, Coefficient of determination; Acc, Accuracy; CNN, Convolutional neural networks; LSTM, Long short term memory.

Acoustic impulse-response and ultrasonic techniques established the feasibility of monitoring tomato softening without destructive penetration. Schotte et al. (1999) derived a stiffness factor from the first resonance frequency and fruit mass and reported a relationship between this objective indicator and firmness evaluated by market experts. On this principle, Aliu et al. (2015) monitored Melody tomatoes over 11 days of storage using dominant resonant frequency, firmness coefficient, elasticity coefficient, and integral acoustic intensity. The firmness coefficient progressively decreased from 4017.6 $Kg^{\frac{2}{3}}\cdot S^{-2}$ to 1458.6 $Kg^{\frac{2}{3}}\cdot S^{-2}$, while the dominant resonant frequency showed particularly pronounced storage-related changes. These results support acoustic impulse response for repeated monitoring of tomato softening, although differences among acoustic parameters and their dependence on storage and biological conditions should be considered when interpreting firmness. Ultrasound provides related mechanical information by characterizing wave propagation through fruit tissues. Mizrach (2007) used ultrasonic probes in contact with greenhouse tomatoes and found that both ultrasonic attenuation and destructively measured firmness decreased during shelf life, with an approximately linear relationship between the two during the softening period. This ultrasonic approaches based on attenuation and propagation velocity for preharvest and postharvest quality evaluation of fresh fruits and vegetables (Mizrach, 2008). These studies demonstrate the feasibility of acoustic and ultrasonic sensing for objective mechanical assessment.

Tactile sensing approaches shift the measurement from global vibration or wave propagation toward local fruit–sensor interaction. He et al. (2024) developed a photometric stereo vision-based tactile sensor that reconstructed the three-dimensional deformation produced when the sensor contacted cherry tomatoes. Deformation-derived features were associated with reference firmness measurements and were subsequently used to monitor firmness changes during storage. This approach converts small surface deformation into a spatially resolved mechanical representation rather than relying only on a single force value. Lin et al. (2023) further integrated vision and tactile sensing into a compliant soft gripper and processed the gripper interaction data using an attention-based CNN–LSTM model. For tomatoes, the reported firmness prediction reached an R² of 0.795. More recent designs have further integrated sensing and manipulation. Liu et al. (2025b) embedded flexible hydrogel pressure sensors in a tomato harvesting gripper and combined tactile sequences with fruit deformation for ripeness classification. At a more integrated level, Mishra et al. (2026) combined vision, tactile, curvature, distance, and pressure sensing within a five-finger soft manipulator for fruit picking, illustrating a transition from isolated firmness measurement toward multimodal perception during harvesting.

Mechanical and tactile methods provide information that is difficult to obtain directly from conventional optical sensing because firmness is fundamentally associated with tissue mechanics. Acoustic and ultrasonic methods offer relatively simple, objective assessment of global mechanical response, whereas vision, tactile and flexible sensors provide localized deformation and force information and can be integrated directly into robotic manipulation. The recent progression from standalone tactile sensors to sensorized soft grippers and multimodal manipulators indicate that the operational focus lies in obtaining mechanical information during the normal harvesting interactions (Lin et al., 2023; Liu et al., 2025a). Future research should consider addressing the sensor errors caused by complex environments and accidental touches, as well as enhancing the long-term stability of the sensors (Mishra et al., 2026).

### 4.3 Electrical and emerging sensing techniques

Electrical sensing provides an additional information source for nondestructive tomato assessment by characterizing the response of fruit tissues to an applied electrical field. During ripening and postharvest storage, changes in ionic mobility, water distribution, and tissue composition can alter resistance, reactance, capacitance, and other frequency-dependent electrical properties (Ziani et al., 2025). These methods differ fundamentally from optical sensing because their signals arise from electrical conduction and polarization, gas-sensor interactions, or field perturbations associated with fruit composition and structure. Applications of electromagnetic and optical techniques in non-destructive quality monitoring of tomatoes are summarized in **Table 3**.

**Table 3:** Applications of electrical and emerging sensing techniques in non-destructive quality monitoring of tomatoes.

Technology	Sample type	Parameters	Algorithm	Accuracy	References
EIS	Hybrid 908	Maturity grading	Basic mathematical calculations	Acc = 88.3%	(Li et al., 2019)
EIS	——	Maturity grading	SVM-RF-GTO	Acc = 97.26%	(Zhang et al., 2024)
EIS	Dongfeng 199	vitamin C	LSTMAE–XGBoost	R^2^ = 0.805	(Wang et al., 2025a)
		SSC		R^2^ = 0.945	
		soluble protein		R^2^ = 0.838	
		TA		R^2^ = 0.845	
Capacitive sensor	Plum tomato	Ripening stage prediction	Logarithmic Function	R^2^ = 0.9919	(Kim et al., 2023)
Electronic Nose	——	Quality classification	WOA-SVM	Acc =99.81%	(Zhou et al., 2023)
Electronic Nose	——	Ripening stage	LeNet	Acc = 86%	(Padilla et al., 2024)
Capacitive sensor+Magnetic induction tomography	——	Freshness prediction	DNN	Acc = 97%	(Chen et al., 2025)
		TA		R^2^ = 0.849	

ELS, Electrical impedance spectroscopy; SSC, Soluble solid content; TA, Titratable acid; Acc, Accuracy; R^2^, Coefficient of determination; SVM, Support vector machine; RF, Random forest; LSTMAE, Long Short-Term Memory Autoencoder; XGBoost, Extreme Gradient Boosting; WOA-SVM, the support vector machine model characterized by whale optimization algorithm; DNN, Deep neural network.

Electrical impedance spectroscopy (EIS) has provided some of the clearest tomato-specific evidence within this category. Li et al. (2019) measured intact fruits from 1 Hz to 1 MHz and identified 100 Hz, 1 kHz, and 1 MHz as informative frequencies for maturity assessment. Impedance, phase angle, resistance, reactance, and capacitance increased with ripening, and selected electrical features classified immature and mature fruit with an accuracy of 88.3%. The study also developed a constant-phase-element equivalent circuit and related electrical changes to soluble solids and pH, indicating that impedance responses reflect a combination of structural and compositional change. This principle was extended by Zhang et al. (2024), who constructed a Double-R-Cole model that explicitly considered contact impedance and combined fitted electrical parameters with biological variables and storage time. Their classifier separated four maturity stages with 97.26% accuracy, demonstrating that equivalent-circuit parameters can provide a more structured representation of postharvest electrical changes than raw impedance alone. Wang et al. (2025a) moved beyond maturity classification and used nine electrical parameters measured over 0.1–1000 kHz to predict vitamin C, soluble sugar, soluble protein, and titratable acidity in 300 tomatoes. A complementary engineering direction was demonstrated by Kim et al. (2023), who developed a capacitive sensing system with LoRa communication for real-time monitoring of plum tomato ripening over two weeks, illustrating the feasibility of low-power, long-distance sensing for continuous postharvest or on-plant monitoring.

Electronic nose systems provide a different sensing mechanism by recording the collective responses of gas-sensor arrays to ripening- and storage-related volatile profiles. Zhou et al. (2023) developed a portable e-nose system for tomato storage-quality prediction and monitored sensor responses over 21 days. The results demonstrated that volatile-response patterns can track storage-related changes using a compact sensing platform. Deep learning has also been introduced to reduce dependence on manually designed gas-sensor features. Padilla et al. (2024) applied a LeNet-5 architecture to E-nose data for ripe identification, with the best configuration achieving a mean training score of approximately 0.95. These studies indicate that e-nose sensing can complement electrical and optical methods by targeting a different information domain, namely volatile metabolism. However, its performance is strongly dependent on sensor composition, temperature, humidity, baseline drift, and calibration stability. Therefore, although high classification accuracy can be achieved under experimental conditions, it cannot be directly concluded that it has the same performance when deployed in a long-term field setting.

Emerging sensing techniques may further broaden the accessible information, although their maturity for tomato quality assessment remains limited. Chen et al. (2025) proposed magnetic induction tomography for quantitative evaluation of internal physical and chemical indicators in fruits and vegetables, using changes in induced electromagnetic fields to reconstruct conductivity-related internal information. The method is attractive because it does not rely on optical penetration and may therefore provide a complementary route to internal quality characterization, but current evidence is still at an early stage and broader fruit-specific validation is require. Moreover, the increasing integration of AI with advanced sensing and spectroscopic platforms has improved the ability to analyze high-dimensional signals (Ziani et al., 2025).

Electrical and emerging sensing techniques offer information that is complementary to optical and mechanical measurements. Impedance and capacitance reflect tissue electrical properties, E-nose systems characterize volatile-response patterns, and magnetic induction approaches aim to probe internal conductivity distributions. The main challenge currently faced is to transfer the concept from the laboratory setting to the field environment, ensuring its transferability and stability in measurement.

### 4.4 Comparison of technologies

The nondestructive techniques reviewed above differ primarily in the physical information they acquire and the quality attributes for which they are most appropriate. **Figure 6** summarizes the characteristics of these technologies. RGB imaging provides rapid and low-cost assessment of spatially resolved external characteristics, but its sensitivity to internal physicochemical changes is inherently limited (Filho et al., 2024). Point spectroscopy offers greater compositional sensitivity with comparatively simple acquisition, whereas multispectral and hyperspectral imaging combines spectral and spatial information and is therefore better suited to heterogeneous quality features, although at substantially higher hardware and data processing cost (Sun et al., 2024). Fluorescence imaging provides excitation dependent physiological information that can remain informative when visible color changes are limited, while acoustic, ultrasonic, and tactile methods directly or indirectly characterize tissue mechanics and are therefore particularly relevant to firmness-related quality. Electrical impedance and electronic-nose systems further extend sensing toward tissue electrical properties and volatile profiles, respectively (Huang et al., 2025a; Liu et al., 2025a).

**Figure 6:**
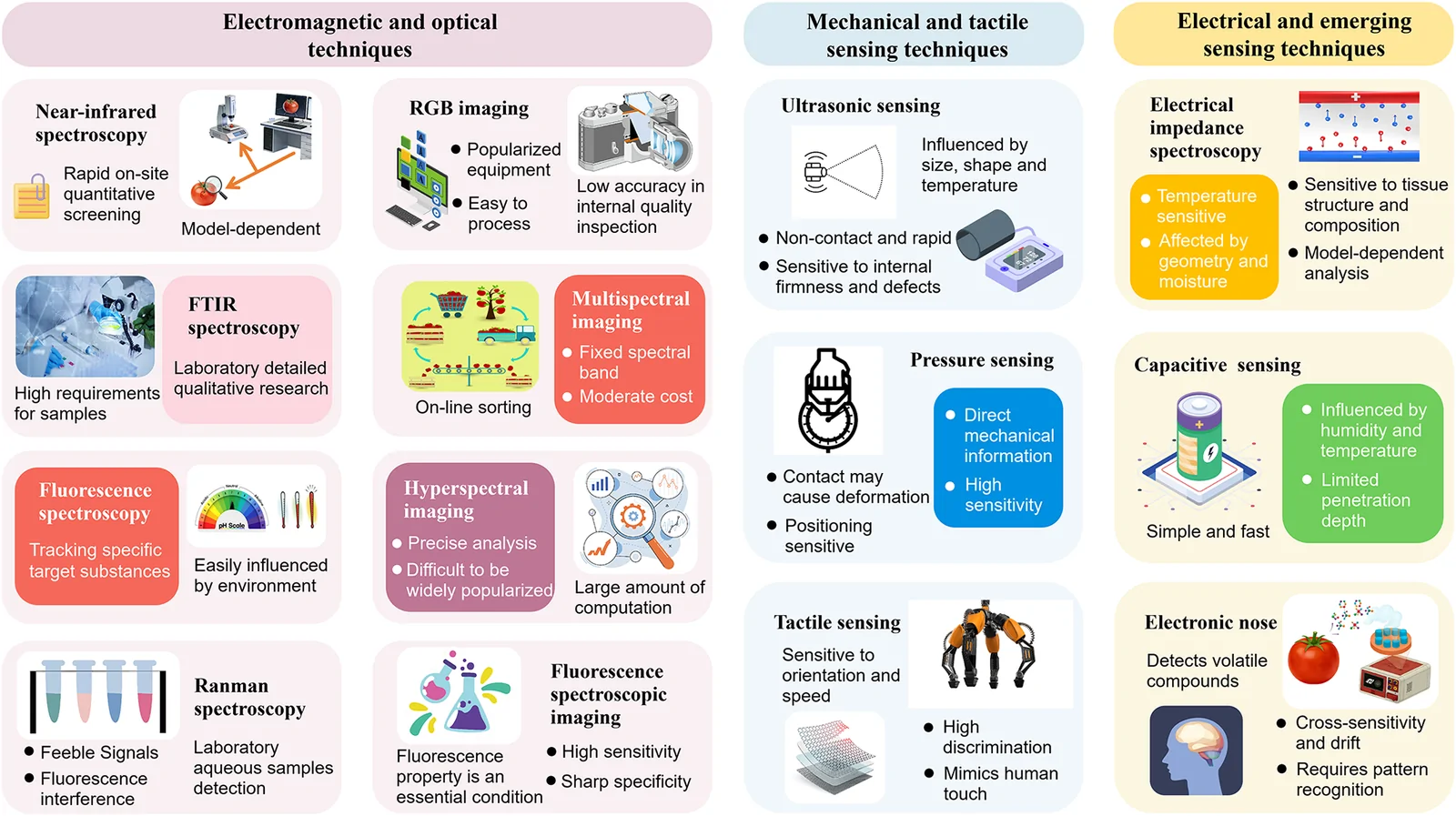
Summary of the characteristics of non-destructive technologies for tomato quality evaluation.

These complementary information domains are accompanied by different practical constraints. RGB and selected point-spectroscopic systems are comparatively compatible with rapid sorting or portable deployment, whereas spectral imaging systems remain constrained by instrument cost, calibration requirements, and computational complexity (Sun et al., 2024; Zhang et al., 2025b). Mechanical and tactile techniques provide physically meaningful firmness information but require controlled excitation or contact, making measurements sensitive to fruit geometry, contact conditions, and tissue heterogeneity (Liu et al., 2025b). Electrical sensing methods can access information not directly available from surface imaging, but electrode coupling, environmental conditions, sensor drift, and calibration stability can affect reproducibility. Furthermore, although the combination of modeling algorithms has improved the accuracy of quality prediction, the computing efficiency requirements of agricultural Internet of Things are quite high. Integrating edge computing is an important challenge in the practical application of sensor systems in agricultural scenarios (He et al., 2026).

## 5 Multimodal sensing and data fusion for tomato quality assessment

Tomato quality assessment involves multiple external, internal, mechanical, and physicochemical attributes that are difficult to characterize comprehensively using a single sensing modality. Accordingly, food-quality sensing has increasingly progressed from single-source measurements toward integrated analysis of complementary data (Guo et al., 2024; Li et al., 2026). The effectiveness of such integration depends not only on the number of sensors or variables, but also on the complementarity and reliability of the information they provide. Therefore, the fusion strategy should be considered before discussing specific sensing combinations.

Data fusion can generally be implemented at three levels, as shown in **Figure 3**. Low-level fusion directly concatenates raw or preprocessed data from different sources. This approach preserves a large amount of original information, but may introduce high dimensionality, scale differences, and redundant variables. Intermediate-level fusion first extracts, selects, or compresses features from each source and then combines the resulting representations. It can reduce redundancy while retaining source-specific information. High-level fusion combines the outputs, probabilities, or decisions of independently developed models (Calvini and Pigani, 2022; Tang et al., 2023). In practice, the appropriate fusion level should be selected according to the physical relationship among the sensing sources, the target quality attribute, the degree of data compatibility, and the intended application rather than according to model complexity alone.

From the perspective of sensing principles, fusion can be divided into homogeneous fusion and heterogeneous multimodal fusion (Calvini and Pigani, 2022; Zhang et al., 2025a). Homogeneous fusion combines measurements generated by the same or closely related sensing mechanisms. Heterogeneous fusion integrates physically different sensing modalities. Although both approaches aim to improve prediction, they should not be interpreted as equivalent. Homogeneous sensor fusion primarily enhances the completeness of information within one sensing domain, whereas multimodal fusion seeks to bridge external appearance, chemical composition, tissue mechanics, and other quality-related responses (Tang et al., 2023; Li et al., 2026). By combining responses associated with different physical manifestations of tomato quality, these approaches can improve the assessment of maturity and quality attributes.

### 5.1 Data fusion based on homogeneous sensors

Information fusion among homogeneous or closely related sensors integrates measurements obtained through the same or similar sensing principles. Representative applications in food analysis include fusion across spectroscopic platforms, wavelength ranges, optical acquisition modes, and spectral or spectral-spatial representations (Guo et al., 2024; Strani et al., 2024). Several tomato studies have demonstrated the value of this type of information integration.

Tan et al. (2023) fused Vis-NIR and NIR hyperspectral information for simultaneous prediction of firmness, SSC, and TA in cherry tomatoes. The fusion can be interpreted as a form of complementary spectral-band integration. Vis-NIR information is generally more closely associated with surface color and visible pigment variation, whereas NIR information provides stronger responses related to water, organic compounds, and other chemical constituents. This represents a typical homogeneous fusion strategy because both instruments provide spectral information, although they operate in different wavelength regions. Fusion improved the prediction of these quality attributes relative to individual spectral information, illustrating the benefit of extending effective spectral coverage. The improvement over the individual spectral ranges therefore likely resulted from broader wavelength coverage and the combination of partially complementary chemical information.

Zheng et al. (2024) combined the 200–600 nm reflectance spectra and 600–1100 nm transmittance spectra for SSC prediction. The two measurements were obtained through different optical paths rather than simply through different wavelength ranges. Reflectance spectra mainly describe the interaction of incident light with the fruit surface and shallow tissue layers, whereas transmittance spectra contain information related to light propagation through a larger tissue volume. The fusion of these two optical responses therefore combines surface-sensitive and volume-sensitive information within the same spectral domain. The PLSR model achieved an R^2^ of 0.9653, which was better than the model yielded with only 200–1100 nm reflectance spectra (R^2^ = 0.8895) and the model yielded with only 600–1100 nm transmittance spectra (R^2^ = 0.9494). Nevertheless, the effectiveness of this approach depends on accurate spectral preprocessing and instrument calibration, since differences in optical geometry and signal intensity can introduce systematic variation.

The fusion methods used in these two studies can be understood mainly as low or intermediate level spectral fusion. In the case of Tan et al. (2023), the spectral information from different instruments was combined to increase the available wavelength information, after which multivariate models were used for quality prediction. In the study of Zheng et al. (2024), reflectance and transmittance spectra were integrated after spectral processing and feature optimization. Such strategies retain the physicochemical information contained in the original spectra, but they also increase the number of variables and the possibility of redundancy. Therefore, variable selection, dimensionality reduction, and comparison with optimized single-instrument models are important for determining whether the additional instrument contributes genuinely complementary information.

Homogeneous fusion based on different spectral instruments or configurations is advantageous because the data sources often share similar acquisition principles, calibration procedures, and physicochemical interpretations (Guo et al., 2024). However, the similarity among the sensing sources also creates a risk of redundancy. A higher prediction accuracy after fusion does not necessarily indicate that each instrument provides independent information. Therefore, source ablation, wavelength selection, redundancy analysis, external validation, and comparison with single-instrument models are required to verify the actual contribution of each spectral source (Xiao et al., 2025). In total, homogeneous fusion is therefore most appropriate when the additional spectral source provides a clearly identifiable information gap that cannot be adequately captured by the primary instrument.

### 5.2 Data fusion based on heterogeneous sensors

Heterogeneous multimodal fusion integrates sensing sources based on different physical principles. Unlike homogeneous spectral fusion, heterogeneous fusion combines measurements that describe different manifestations of tomato quality. Imaging can characterize external color, shape, size, and texture; spectroscopy can provide information related to chemical composition and tissue optical properties; acoustic and tactile sensing can reflect firmness and mechanical structure; and electronic sensors can respond to volatile, electrical, or other physicochemical properties (Calvini and Pigani, 2022; Tang et al., 2023; Li et al., 2026). The purpose of heterogeneous fusion is therefore to establish a more comprehensive representation of quality by combining physically complementary information.

Baltazar et al. (2008) provided an early example by combining colorimeter measurements with acoustic impact responses for tomato maturity classification using a Bayesian classifier. The colorimeter described external color changes associated with ripening, whereas the acoustic response reflected changes in tissue structure and firmness. The multisensor model reduced the classification error from 48% for the univariate case to 11%. This improvement can be attributed to the complementary nature of the two modalities. The use of a Bayesian classifier also represents a form of high-level or decision-oriented fusion, in which information from different sensing sources is incorporated into a probabilistic classification framework. This study demonstrates that heterogeneous fusion can be effective when external appearance and internal mechanical condition do not change synchronously.

Liu et al. (2024b) further combined RGB imaging, Vis-NIR spectroscopy, and haptic sensing for tomato maturity assessment. RGB imaging captures external color and appearance, Vis-NIR spectroscopy provides information related to chemical composition and tissue optical properties, and haptic sensing reflects mechanical changes related to firmness. The study used feature-level fusion to combine the representations extracted from the three modalities before classification. Compared with the individual sensing sources, the fused model improved maturity classification, particularly for samples showing inconsistent external and internal ripening. The effectiveness of this strategy can be explained by the complementary information structure. The fusion model can distinguish samples that appear similar externally but differ in internal maturity. However, feature-level fusion also requires careful normalization and alignment because the three data types differ substantially in dimensionality, scale, and noise characteristics.

More heterogeneous fusion has increasingly incorporated image and spectral information. Elmetwalli et al. (2024) combined RGB indices and spectral reflectance indices with machine learning models to estimate several biochemical and physical attributes of tomato and mandarin fruit. Zheng et al. (2025) more explicitly fused 200–1100 nm spectra with color and texture features of RGB images for cherry tomato lycopene prediction, obtaining an R^2^ of 0.95 and demonstrating that surface image information could complement weak spectral signatures associated with relatively low lycopene concentrations. Related work has integrated hyperspectral feature wavelengths with color features and improved 1D-CNN models for tomato ripeness detection (Zhou et al., 2026). Deep fusion extends this approach beyond direct feature concatenation. TomatoRipen-MMT uses separate RGB and NIR encoders with bidirectional cross spectral attention to learn interactions between modality-specific representations (Dubey et al., 2025). The model achieved 94.8% classification accuracy on a dataset derived from 224 hyperspectral samples, outperforming the reported single modality and simpler fusion baselines. Although promising, the relatively limited dataset and derivation of both RGB and NIR inputs from the same hyperspectral cubes mean that broader claims of cross-sensor robustness require independent acquisition and external validation.

The success of heterogeneous multimodal fusion depends on whether the sensing sources provide physically complementary information. The examples mentioned above show that fusion methods have evolved from probabilistic decision integration and feature concatenation toward learned cross-modal representation. However, increased model complexity does not automatically guarantee better information integration. Independent validation and analysis of modality contributions are necessary to determine whether the model learns stable physical relationships or merely exploits dataset-specific correlations (Li et al., 2026).

In the future, the selection of multimodal sensing modes for tomato quality detection should be guided by measurable complementarity and the target quality attributes. The models should conduct ablation experiments of a specific pattern to quantify the contribution of each sensor data. The missing mode should also be taken into consideration, as the actual system cannot assume that all sensors remain equally reliable throughout their operation. Moreover, interpretability, calibration transfer, computational efficiency, synchronization, and hardware cost should be evaluated together with predictive performance (Xiao et al., 2025; Li et al., 2026). Multimodal sensing is most valuable when complementary sensors reduce the information gaps inherent to individual modalities.

## 6 Conclusion and perspective

This review systematically summarizes recent progress in nondestructive tomato quality assessment from conventional single sensor measurements to multidimensional sensing and data fusion. Point spectroscopy offers greater sensitivity to compositional changes with relatively simple instrumentation, whereas RGB imaging provides rapid and economical characterization of color, morphology, texture, and external defects. Hyperspectral and multispectral imaging further integrate spectral and spatial information but involve greater hardware, calibration, and computational requirements (Sun et al., 2024). Fluorescence and Raman techniques provide complementary physiological or molecular information, while terahertz methods extend sensing toward dielectric and moisture-related responses, although their practical technical maturity differs considerably (Fu et al., 2024; Jayan et al., 2025). Mechanical, acoustic, tactile, electrical, and volatile sensing provide additional information on firmness, tissue electrical properties, and metabolic changes that cannot be fully represented by optical measurements.

RGB imaging is well suited to rapid evaluation of external appearance, while point spectroscopy and spectral imaging provide increasingly detailed information on internal composition and spatial heterogeneity. Fluorescence imaging offers complementary sensitivity to physiological changes. Acoustic, ultrasonic, and tactile sensing are more directly related to tissue mechanical properties such as firmness. Electrical impedance and electronic nose systems further extend sensing toward tissue electrical characteristics. These techniques provide complementary information on appearance, composition, physiological status, mechanical properties, and other quality characteristics. Their applicability depends on the target attribute, sensing principle, acquisition conditions, and validation strategy. Current research is progressing from single sensor assessment toward integrated sensing frameworks that combine multiple information sources for more comprehensive tomato quality evaluation. Multimodal sensing can reduce information gaps by combining complementary modalities. It is important to note that its value depends on the individual contributions of the information, rather than the number of sensors used (Li et al., 2026). Nevertheless, most reported systems remain strongly dependent on controlled datasets and experimental conditions, leaving substantial gaps in cross-domain robustness, system integration, and industrial validation.

Future research should prioritize integrated data strategies and model generalization rather than further optimization within individual datasets. The selection of fusion strategies should be guided by measurable complementarity and target quality attributes, and a balance should be struck between information retention, computational requirements, and interpretability (Guo et al., 2024; Strani et al., 2024). Deep learning and hyperspectral reconstruction may reduce manual feature engineering or hardware requirements, but they remain dependent on representative training data and rigorous validation (Ahmed et al., 2025b; Ding et al., 2025). Model assessment should move from random splitting of samples collected under similar conditions toward genuinely independent evaluation across cultivars, seasons, and postharvest environments (Fearn et al., 2025). Long term use also requires explicit strategies for dataset shift, calibration transfer, and model maintenance because instrument, temperature, orientation, and environmental changes can alter sensor responses (Roger et al., 2024; Fan et al., 2025; Liu et al., 2025a). Explainable AI can assist in identifying influential wavelengths, image regions, and sensor variables, but such explanations should support model diagnosis and validation rather than be interpreted as causal evidence (Arrighi et al., 2026).

Industrial implementation should focus on integrated and maintainable monitoring systems matched to specific tomato quality decisions. RGB or multispectral imaging can provide high-throughput external screening, while compact spectral sensors can be incorporated when information on internal composition provides sufficient additional value. Recent smartphone spectrometers illustrate the feasibility of substantial reductions in sensor size and cost, while AI assisted nondestructive testing is increasingly directed toward portable devices, edge computing, transferable models, and automated decision-making (Rayhan et al., 2025; Yu et al., 2025). Broader photonics roadmaps similarly identify miniaturization, sensor integration, and intelligent processing as important directions for future systems (Smeesters et al., 2025). However, industrial adoption will require standardized acquisition protocols, robust reference methods, calibration transfer across instruments, interlaboratory validation, and closer alignment between research models and analytical standards (Cozzolino, 2025; Shi et al., 2026). Beyond isolated sorting decisions, sensor measurements could ultimately be linked with storage history and environmental data through validated digital-twin frameworks to support shelf life and supply chain management (Defraeye et al., 2021). Thus, future progress will depend less on marginal improvements in laboratory prediction accuracy than on developing transferable, interpretable, standardized, and economically viable tomato quality monitoring systems.
